# Quantitative Transcriptomic and Proteomic Analysis of Fruit Development and Ripening in Watermelon (*Citrullus lanatus*)

**DOI:** 10.3389/fpls.2022.818392

**Published:** 2022-03-22

**Authors:** Yongtao Yu, Shaogui Guo, Yi Ren, Jie Zhang, Maoying Li, Shouwei Tian, Jinfang Wang, Honghe Sun, Yi Zuo, Yakun Chen, Guoyi Gong, Haiying Zhang, Yong Xu

**Affiliations:** National Watermelon and Melon Improvement Center, Beijing Academy of Agriculture and Forestry Sciences, Key Laboratory of Biology and Genetic Improvement of Horticultural Crops (North China), Beijing Key Laboratory of Vegetable Germplasm Improvement, Beijing, China

**Keywords:** watermelon, proteomics, transcriptomics, comparative analysis, fruit ripening

## Abstract

Fruit ripening is a highly complicated process, which is modulated by phytohormones, signal regulators and environmental factors playing in an intricate network that regulates ripening-related genes expression. Although transcriptomics is an effective tool to predict protein levels, protein abundances are also extensively affected by post-transcriptional and post-translational regulations. Here, we used RNA sequencing (RNA-seq) and tandem mass tag (TMT)-based quantitative proteomics to study the comprehensive mRNA and protein expression changes during fruit development and ripening in watermelon, a non-climacteric fruit. A total of 6,226 proteins were quantified, and the large number of quantitative proteins is comparable to proteomic studies in model organisms such as *Oryza sativa* L. and *Arabidopsis*. Base on our proteome methodology, integrative analysis of the transcriptome and proteome showed that the mRNA and protein levels were poorly correlated, and the correlation coefficients decreased during fruit ripening. Proteomic results showed that proteins involved in alternative splicing and the ubiquitin proteasome pathway were dynamically expressed during ripening. Furthermore, the spliceosome and proteasome were significantly enriched by Kyoto Encyclopedia of Genes and Genomes (KEGG) pathway enrichment analysis, suggesting that post-transcriptional and post-translational mechanisms might play important roles in regulation of fruit ripening-associated genes expression, which might account for the poor correlation between mRNAs and proteins during fruit ripening. Our comprehensive transcriptomic and proteomic data offer a valuable resource for watermelon research, and provide new insights into the molecular mechanisms underlying the complex regulatory networks of fruit ripening.

## Introduction

Watermelon (*Citrullus lanatus*, 2n = 2× = 22) belongs to the *Citrullus* genus of the Cucurbitaceae family, originated in Africa and is an important cucurbit crop grown throughout the world. Watermelon has emerged as an important experimental model to study the molecular mechanisms of non-climacteric fruits ripening (Erickson et al., 2005). This reflects its economic value and many favorable genetic characteristics, such as a relatively short life cycle, smaller genome size (359.8 Mb), diploid cultivar and stable genetic transformation (Guo et al., 2013). Ripening of fleshy fruits is a highly complex process that involves dramatic changes in sugar content, fruit color, fruit texture, flavor, and aroma (Giovannoni, 2001; Seymour et al., 2013). Depending on the ethylene system II, fleshy fruits are physiologically classified as climacteric fruits and non-climacteric fruits. System I is known as ethylene autoinhibitory and ethylene is produced in vegetative tissues including young fruit, while system II produces greater ethylene during climacteric fruit ripening and is autocatalytic. Climacteric fruits contain System I and System II, whereas non-climacteric fruits only produce ethylene by System I (Liu et al., 2015; Yue et al., 2020). It has long been thought that the ripening of climacteric and non-climacteric fruits is regulated by ethylene and abscisic acid (ABA), respectively (Alexander and Grierson, 2002; Li et al., 2011; Pech et al., 2012; Jia et al., 2013a; Leng et al., 2014; Wang et al., 2017b). In the last 20 years, much progress has been made toward understanding the complicated molecular mechanisms for ethylene-modulated ripening in climacteric fruits, and some key signaling components, including ethylene receptors [LeETHYLENE RECEPTOR 1 (LeETR1), LeETR2, NEVER RIPE (NR), LeETR4, LeETR5, LeETR6, and LeETR7] and several transcription factors [RIPENING INHIBITOR (RIN), COLORLESS NON-RIPENING (CNR), and NON-RIPENING (NOR)], have been identified, which significantly deepen our understanding of the molecular mechanisms of ripening from primary signal perception events to downstream genes expression (Vrebalov et al., 2002; Adams-Phillips et al., 2004; Manning et al., 2006; Klee and Giovannoni, 2011; Yuan et al., 2016; Quinet et al., 2019; Chen et al., 2020). Over the past decade, some ABA signaling components have been reported to be involved in regulation of ripening in non-climacteric fruits (Chai et al., 2011; Jia et al., 2011, 2013b; Han et al., 2015). However, the complex mechanisms and regulatory networks of non-climacteric fruits ripening are still largely unknown.

Due to rapid advances in high-throughput sequencing technologies, multi-omic studies have dramatically promoted the dissection of the molecular mechanisms of fruit ripening, such as transcriptomics, proteomics, and metabolomics (Belouah et al., 2019; Umer et al., 2020). Wu et al. (2014a), Li et al. (2015), and Wang et al. (2017a) conducted transcriptomic and proteomic analyses of mango, pear, and citrus during fruit development and ripening, and 2,754, 1,810, and 4,648 proteins were identified or quantified, respectively (Wu et al., 2014a; Li et al., 2015; Wang et al., 2017a). Wu et al. (2014b) performed an integrative analysis of the transcriptome and proteome in the pulp of a spontaneous late-ripening sweet orange mutant and its wild type. A total of 1,839 proteins were identified, and they found that a number of genes displayed inconsistency at the transcript and protein levels (Wu et al., 2014b). Belouah et al. (2019) conducted transcriptomic and proteomic analysesof nine developmental stages in tomato, and a total of 2,375 proteins were quantified. Furthermore, they found that mRNAs and proteins had a poor correlation during ripening (Belouah et al., 2019). In addition, comparisons of mRNA levels, protein abundances and enzymatic activities have revealed low correlations between the metabolome and transcriptome, indicating that transcriptomics is not sufficient to understand protein dynamics (Gibon et al., 2006; Wienkoop et al., 2008). However, a more direct correlation is expected for proteins and metabolites (Wienkoop et al., 2008), making quantitative proteomics to be a powerful approach for establishing functional correlations between phenotypes and genotypes, and characterizing biochemical networks (Palma et al., 2011; Li et al., 2015).

Although previous studies have conducted comparative analyses of the transcriptome and proteome during fruit development and ripening, most studies have the phenomenon of smaller amounts of quantified proteins or samples or biological replicates. In this study, we carried out high-throughput RNA sequencing (RNA-seq) and quantitative proteomics of four critical developmental stages in watermelon (three repeats for each stage), and quantified 18,856 genes and 6,226 proteins. The inclusion of more quantitative mRNAs and proteins is more conducive to conduct comparative analyses, which can better reflect the dynamic changes in mRNA-protein correlations during fruit ripening.

## Materials and Methods

### Plant Materials and Sugar Content Measurement

**FIGURE 1:**
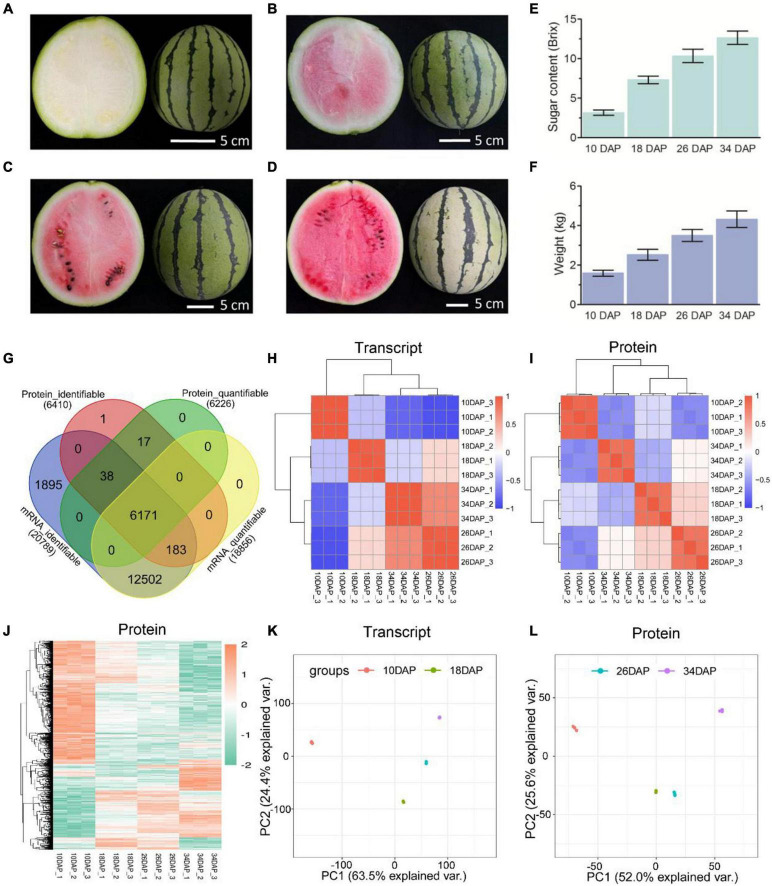
Overview and reproducibility of the proteome and transcriptome. Fruits of the cultivated watermelon 97103 at four different stages of ripening. **(A)** White [10 days after pollination (10 DAP)], **(B)** white-pink (18 DAP), **(C)** red flesh (26 DAP), **(D)** red-ripe (34 DAP). Sugar content of center flesh **(E)** and weight **(F)** statistics of four stages. **(G)** Venn diagram of identifiable and quantifiable mRNAs and proteins in this study. Heatmaps of Pearson correlation values of the TMT-labeled protein **(I)** and mRNA **(H)** abundances of each of the replicates compared to every other replicate. **(J)** Hierarchical clustering analysis of differentially expressed proteins (DEPs). Principal component analysis (PCA) was used to evaluate the reproducibility of the samples from proteome **(L)** and transcriptome **(K)**.

Watermelon *C*. *lanatus* (Thunb.) Matsum. & Nakai subsp. *vulgaris* cv 97103 was used in this study. In order to make the fruit grow uniformly, we keep only one watermelon per plant. Flowers were hand-pollinated at 3–5 nodes and tagged. Transcriptomic and proteomic analyses were performed on three biological replicates of watermelon center flesh samples collected at four developmental stages [10,18, 26, and 34 DAP (days after pollination), Figures 1A–D], each replicate resulting from the pooling of at least 10 fruits. Samples were rapidly frozen in liquid nitrogen and stored at –80°C. Degrees Brix were measured using a pocket refractometer (model pal-1; ATAGO) from a sample of juice collected from the center of each watermelon.

### Total RNA Extraction, RNA-Sequencing Library Construction, Sequencing, and Data Analysis

Total RNA was isolated with a Total RNA Rapid Extraction Kit (Huayueyang Biotechnologies Co. Ltd., Beijing, China). Three biological replicates were carried out for RNA-seq. The RNA-seq libraries were generated using the NEBNext Ultra™ RNA Library Prep Kit, and were sequenced on the Illumina NovaSeq 6000 system using 150 PE mode. The clean reads were aligned to watermelon reference genome^1^ using Tophat v2.0.12 (Trapnell et al., 2009). HTSeq v0.6.125 was used to count the read numbers mapped to each gene (Anders et al., 2015). The fragments per kilobase of exon per million mapped fragments (FPKM) was calculated based on the gene length and read count mapped to that gene. Genes with FPKM > 0.3 were considered as expressed genes. The DESeq package (1.18.0) was used to analyze the DEGs (Anders and Huber, 2010). Genes with padj < 0.05 and log_2_FoldChange > 1 were considered as differentially expressed genes (DEGs). *P-*values were adjusted using the Benjamini–Hochberg’s approach for controlling the false discovery rate (Benjamini and Hochberg, 1995).

### Reverse Transcription-Quantitative PCR

Two micrograms of total RNA was subjected to first-strand cDNA synthesis using a Roche Transcriptor First Strand cDNA Synthesis Kit and an oligo (dT18) primer. Reverse transcription-quantitative PCR (RT-qPCR) was performed with SYBR Premix Ex Taq (Takara, Dalian, China) using a BioRad Real-Time System CFX96TM C1000 Thermal Cycler (Singapore). The *ACTIN7* gene was used as an internal control. The primers for RT-qPCR are listed in Supplementary Table 1. For all RT-qPCR analyses, the assays were repeated three times, and the means of four biological experiments were used to estimate gene expression.

### Protein Extraction, Trypsin Digestion, and Tandem Mass Tag Labeling

Samples were ground in liquid nitrogen, and then the powder was transferred to 5 mL clean tubes and sonicated by an ultrasonic processor (Ningbo Scientz Biotechnology Co., Ltd., Ningbo, China) in lysis buffer [5 mM DTT, 0.5% Triton X-100, 50 μM PR-619, 3 μM TSA, 50 mM NAM, 2 mM EDTA, 0.5 mM PMSF, 1X protease inhibitor (Roche, Mannheim, Germany)] on ice. Then the same volume of tris-saturated phenol (pH 8.0) was added to the sonicated samples, and vortexed for 5 min. After centrifugation (4°C, 20 min, 8,000 rpm), the upper phase was transferred to a new tube. Five volumes of ammonium sulfate-saturated methanol were added to the samples, which were then mixed well and stored at –20°C for 8 h. After centrifugation (4°C, 20 min, 8,000 rpm), the precipitate was washed once with ice-cold methanol and twice with acetone. We used 8 M urea to redissolve the proteins, and the protein concentration was determined by a BCA kit (Thermo Fisher Scientific, Waltham, MA, United States). Trypsin digestion and TMT labeling were conducted according to a previously published method (Wang et al., 2019). Briefly, the same amount of protein from each sample was used for digestion. An appropriate amount of the standard protein was added, and the volume was adjusted to the same with lysis buffer. TCA was added slowly to a final concentration of 20%, mixed with vortex, and precipitated at 4°C for 2 h. After centrifugation (4°C, 5 min, 4,500 *g*), discarded the supernatant and washed the precipitate 2–3 times with precooled acetone. After the precipitate was dried, TEAB was added to a final concentration of 200 mM. The precipitate was dispersed by ultrasound. Trypsin was added to the samples at the ratio of 1:50 (trypsin/protein, m/m) for digestion overnight. Samples were reduced with 5 mM DTT for 30 min at 56°C. After that, Iodoacetamide (IAA) was added to a final concentration of 11 mM, and incubated at room temperature for 15 min in the darkness.

### HPLC Fractionation, LC-MS/MS Analysis, and Database Search

The tryptic peptides were fractionated through high pH reverse-phase HPLC using Thermo Betasil C18 column (5 μm particles, 10 mm ID, 250 mm length). Then the peptides were separated into 60 fractions by a gradient of acetonitrile (8–32%, pH 9.0). Finally, the peptides were combined into 6 fractions and dried by a vacuum-type centrifuge. LC-MS/MS Analysis and Database Search were conducted according to the previously published method (Huang et al., 2020). Briefly, we subjected the peptides to an NSI source, followed by MS/MS in Q ExactiveTM (Thermo Fisher Scientific, Waltham, MA, United States) coupled with UPLC online. Maxquant search engine (v.1.5.2.8) was used to process the resulting MS/MS data. Tandem mass spectra was searched with the uniprot database. Trypsin/P was specified as cleavage enzyme, and we allowed up to four missing cleavages. About 20 ppm was set for the mass tolerance for precursor ions in First search, and 5 ppm was used in Main search. About 0.02 Da is set for the mass tolerance for fragment ions. FDR was adjusted to <1%.

### Data Analysis

The definition of up-regulated differentially expressed protein (DEP) in this study is *P* value < 0.05 and fold change is more than 1.3, whereas those with a (FC) < 1/1.3 (*P* value < 0.05) were considered as down-regulated proteins. The Gene Ontology (GO) annotations for the proteome were derived from the UniProt-GOA database.^2^ Identified protein IDs were converted to UniProt IDs, and then mapped to GO IDs. If the identified proteins did not exist in the UniProt-GOA database, the alignment method (InterProScan soft, United States) was employed for the GO functional annotation of the protein. Proteins were classified into three categories by GO annotation: Cellular Compartment (CC), Molecular Function (MF), and Biological Process (BP). For each category, a two-tailed Fisher’s exact test was used to test the enrichment of the DEPs. A corrected *P* value < 0.05 was considered significant for GO analysis. The Kyoto Encyclopedia of Genes and Genomes (KEGG) database was used to identify enriched pathways. KEGG pathways were classified into hierarchical categories according to the KEGG website.

For further hierarchical clustering based on DEPs functional classification (such as GO, Pathway, Domain, and Complex), we first collated all the categories obtained after enrichment along with their *P* values. Then we included the categories that were enriched in at least one of the clusters with a *P* value < 0.05, and the function x = –log_10_ (*P* value) was used to transform the filtered *P* value matrix. Finally, these x values were z-transformed for each functional category. One-way hierarchical clustering (Euclidean distance, average linkage clustering) was used to cluster z scores in Genesis. Cluster memberships were visualized using the heatmap function of the “gplots” R-package.

For correlation coefficient calculation, we first combined the expression levels of proteins and mRNAs based on the corresponding relationship between protein and mRNA IDs, then we obtained the corresponding expression levels of co-quantified mRNAs and proteins. With these data, scatter plots of protein and mRNA expression levels could be drawn. To unify the definition of expression level, protein and transcript expression was log_2_ transformed before drawing the scatter plot, and the mean value were subtracted. The transformed mean value of the repeated sample was used to draw the scatter plot.

For Gene Set Enrichment Analysis (GSEA), we first calculated the Pearson correlation coefficients of each protein and its corresponding transcript, and tabulated these Pearson correlation coefficients. KEGG annotations were used as a set of genes with known functions, and GSEA was conducted to study the main KEGG pathways of different regulatory relationships using the above correlation coefficients according to a previously published method. The pathways with NOM *P* value < 0.05 were screened to draw the GSEA map (Subramanian et al., 2005; Mertins et al., 2016).

## Results

### Transcriptomic and Proteomic Profiles During Watermelon Fruit Development and Ripening

**FIGURE 2:**
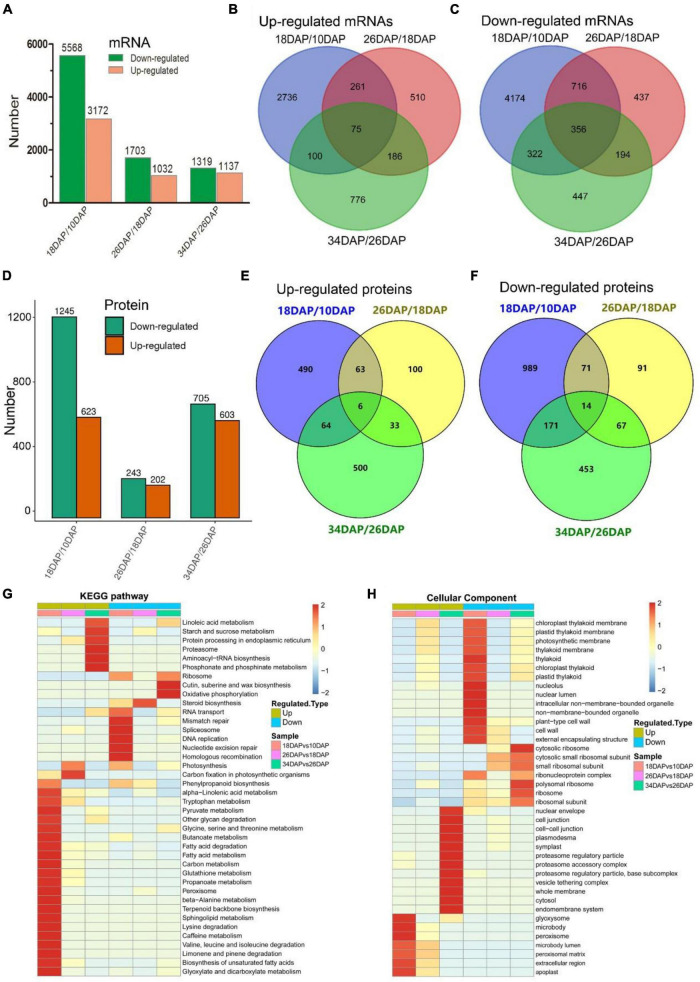
RNA-seq and TMT-labeled quantitative proteomics analyses of watermelon fruit development and ripening. **(A,D)** Differentially expressed genes and proteins statistics of three groups (18 DAP vs 10 DAP, 26 DAP vs 18 DAP, and 34 DAP vs 26 DAP). Venn diagrams of up-regulated mRNAs **(B)**, down-regulated mRNAs **(C)**, up-regulated proteins **(E)**, down-regulated proteins **(F)** in three groups. **(G,H)** Hierarchical clustering analysis of KEGG pathways and GO terms (cellular components) in three groups.

**FIGURE 3:**
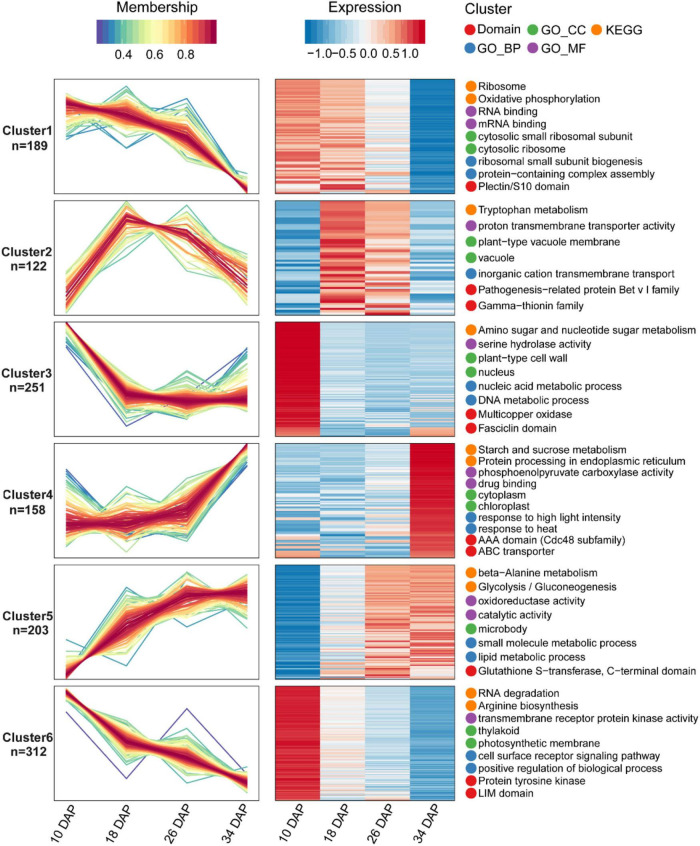
Fuzzy c-means algorithm and enrichment analysis of DEPs. A total of 1,235 DEPs were divided into six clusters by the Fuzzy c-means algorithm, and the top two enriched items from GO/KEGG/Domain enrichment analysis were displayed on the right side of the corresponding clusters.

Samples were collected at four key developmental stages [10 days after pollination (DAP), 18 DAP, 26 DAP, and 34 DAP] (Figures 1A–D), and the sugar content of the center flesh and fruit weight statistics are shown in Figures 1E,F. For RNA-seq, a total of 81.5 million high-quality, clean and mapped reads were obtained (NovaSeq 6000, PE150), and each sample had at least 5.59 million reads. For quantitative proteomics, a total of 1,080,654 spectrums were generated from the TMT experiments. These high-quality transcriptomic and proteomic datasets provided a solid foundation for our comparative analysis. The repeatability analysis of samples from the proteome and transcriptome showed that the results of mRNA and protein quantification were reliable (Figures 1H–L). A total of 18,856 [83.4%, Fragments per kilobase of exon per million mapped fragments (FPKM > 0.3)] genes were quantified in the flesh, and the number of genes covered by our transcriptomic dataset was comparable to that in other studies (Grassi et al., 2013; Guo et al., 2015). Genes with padj < 0.05 and log_2_FoldChange > 1 were considered as DEGs. The differences of transcriptome dynamics at four developmental stages were shown in Figures 2A–C (Supplementary Data 1). We also conducted aRT-qPCR assay to validate our RNA-seq results (Supplementary Figure 1). Additionally, a total of 6,410 and 6,226 proteins were identified and quantified by our proteomic data, respectively (Supplementary Data 2). Proteins with a fold change (FC) > 1.3 (*P* value < 0.05) were assigned as up-regulated proteins, whereas those with a (FC) < 1/1.3 (*P* value < 0.05) were considered as down-regulated proteins. On the basis of these criteria, 623 proteins were up-regulated and 1,245 proteins were down-regulated in 18 DAP vs 10 DAP group, 202 proteins were up-regulated and 243 proteins were down-regulated in 26 DAP vs 18 DAP group, and 603 proteins were up-regulated and 705 proteins were down-regulated in 34 DAP vs 26 DAP group (Figure 2D and Supplementary Data 2). A total of 2,729 DEPs were found in three groups, and 6 and 14 proteins were co-up-regulated and co-down-regulated in three groups, respectively (Figures 2E,F). Using the Fuzzy c-means algorithm, 1,235 DEPs were grouped into six clusters (clusters 1-6), which represented 45.25% of all DEPs (Figure 3 and Supplementary Data 3). Furthermore, the top two Domain, GO, and KEGG pathway enrichment results for the six clusters were shown on the right sideof Figure 3.

We further conducted GO and KEGG pathway enrichment analyses of all DEPs. The up-regulated biological pathways in 18 DAP vs 10 DAP group included starch and sucrose metabolism, glycolysis/gluconeogenesis, citrate cycle (TCA cycle), phenylpropanoid biosynthesis and so on. The down-regulated biological pathways in 18 DAP vs 10 DAP group included cell wall, spliceosome, ribosome and so on (Figures 2G,H and Supplementary Datas 4, 5). The up-regulated biological pathways in 26 DAP vs 18 DAP group were glycoside metabolic process, fatty acid degradation, protein processing in endoplasmic reticulum and so on. The down-regulated biological pathways in 26 DAP vs 18 DAP group were cell wall, plant-type cell wall, ribosome, and so on (Figures 2G,H and Supplementary Datas 6, 7). The up-regulated biological pathways in 34 DAP vs 26 DAP group included starch and sucrose metabolism, proteasome, protein processing in endoplasmic reticulum and so on. The down-regulated biological pathways in 34 DAP vs 26 DAP group contained ribosome, suberine and wax biosynthesis, cytoplasmic mRNA processing body assembly and so on (Figures 2G,H and Supplementary Datas 8, 9).

### Integrative Analysis of the Proteome and Transcriptome

**FIGURE 4:**
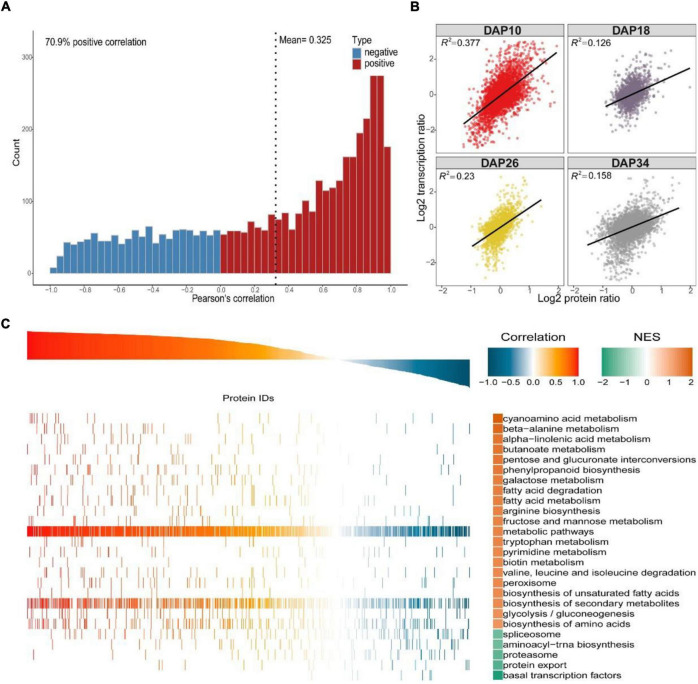
Integrative analysis of the proteome and transcriptome. **(A)** Pearson correlation analysis of overlapping differentially expressed proteins/genes. In total, 70.9% of genes were positively correlated, and the mean Pearson correlation coefficient was 0.325. **(B)** Correlation plots obtained at each of the four stages, and data were log-transformed before analysis. Correlation coefficients (*R*^2^) were given on top of each plot. **(C)** GSEA of global mRNA-to-protein correlation and enrichment analysis of the corresponding gene set.

**FIGURE 5:**
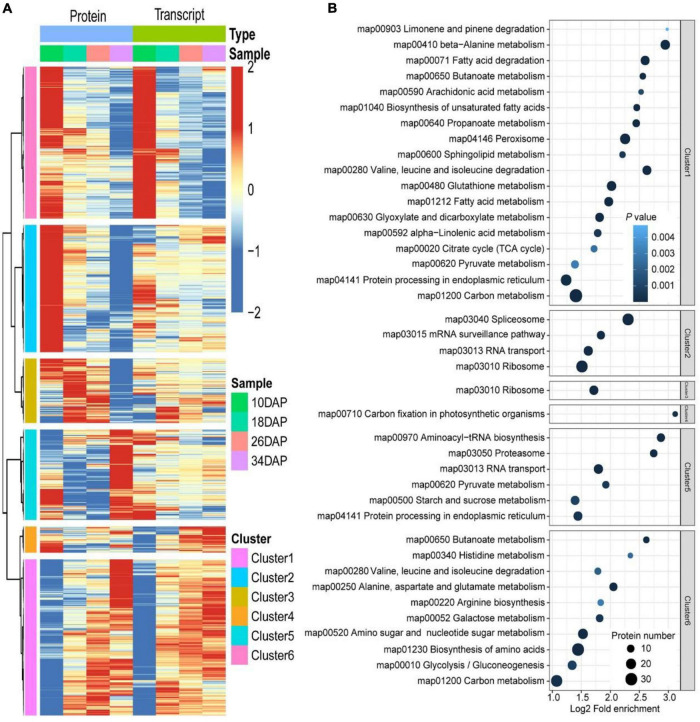
Hierarchical and KEGG pathway enrichment analyses of DEGs and DEPs. **(A)** Hierarchical clustering analysis of 2,365 proteins and their corresponding mRNAs. The expression of proteins and corresponding mRNAs were transformed by log_2_ and subtracted from the mean value, the hclust “ward.D” method was used for cluster analysis. **(B)** Dot plots of enriched KEGG pathways in the six clusters shown in panel **(A)**. The x-axis shows the fold enrichment of each KEGG pathway, where the color denotes the *P* value and the size of the dot denotes the number of IDs assigned to each KEGG pathway.

Considering that protein abundances are not only determined by transcript levels, we conducted a correlation analysis between the transcriptome and proteome during fruit ripening. Firstly, we calculated Pearson correlation coefficients of overlapping mRNAs and proteins. In total, 70.9% of genes were positively correlated, and the mean Pearson correlation coefficient was 0.325 (Figure 4A and Supplementary Data 10). However, we used correlation coefficient to evaluate the correlation between mRNAs and proteins. Secondly, we drew a scatter plot for each period using the log-transformed expression of mRNAs and proteins, and found that correlation coefficients (*R*^2^) decreased during fruit development, indicating that the relationship between mRNAs and proteins weakened during ripening (Figure 4B and Supplementary Data 11). Additionally, Venn diagram of identifiable and quantifiable mRNAs and proteins showed that 99% of the quantifiable proteins overlapped with quantifiable mRNAs (Figure 1G and Supplementary Datas 1, 2). However, we found that many up-regulated and down-regulated proteins were not up-regulated and down-regulated at the mRNA level, respectively (Supplementary Figure 2). These results suggested that post-transcriptional and post-translational mechanisms might play critical roles in regulation of fruit ripening-associated genes expression. Thirdly, we conducted GSEA to study the main KEGG enrichment pathways of different regulatory relationships using the above correlation coefficients. The positively correlated KEGG pathways included phenylpropanoid biosynthesis, fructose and mannose metabolism, galactose metabolism, glycolysis/gluconeogenesis and so on. The negatively correlated KEGG pathways were spliceosome, proteasome, protein export, aminoacyl-tRNA biosynthesis and basal transcription factors (Figure 4C and Supplementary Data 12). Finally, we subjected the transcriptomic and proteomic datasets to hierarchical clustering based on Ward’s minimum variance method (Murtagh and Legendre, 2014; Harvald et al., 2017). A total of 2,365 proteins and their corresponding mRNAs were grouped into six clusters, which further illustrated the correlation between mRNAs and proteins (Figure 5A and Supplementary Data 13). In addition, we conducted KEGG pathway enrichment analysis of the six clusters, and the corresponding enrichment pathways were shown in Figure 5B (Supplementary Data 14).

### mRNA and Protein Expression of a Subset of Ripening-Associated Genes

Previous genome-wide association studies (GWAS) and quantitative trait locus (QTL) studies have identified some important candidate genes, which are associated with watermelon development and ripening (Guo et al., 2015, 2019; Branham et al., 2017; Umer et al., 2020). Here, we examined the expression levels of these genes and those of other genes that might modulate fruit ripening at both mRNA and protein levels, such as ABA and ethylene biosynthesis and signaling-related genes, spliceosome-associated genes and proteasome-related genes.

**FIGURE 6:**
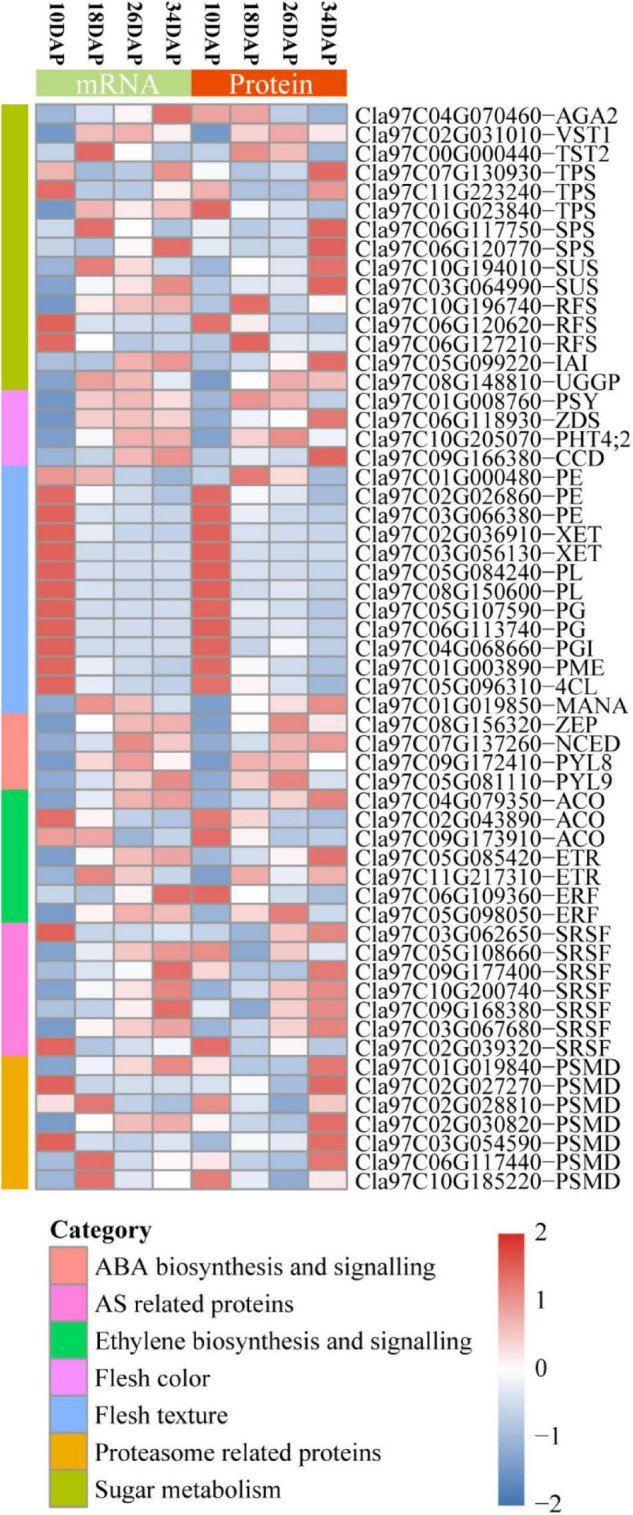
Heatmap for protein and mRNA expression of a subset of fruit ripening-associated genes. The Stat.mean values represent the averaged magnitude, and direction of fold changes at the gene set level corresponds to the color-coded up-regulated (red) and down-regulated (blue) changes. AGA2, α-galactosidase 2; VST1, vacuolar sugar transporter 1; TST2, tonoplast sugar transporter 2; TPS, trehalose-phosphate synthase; SPS, sucrose phosphate synthase; SUS, sucrose synthase; IAI, insoluble acid invertase; RFS, raffinose synthase; UGGP, UDP-Gal/Glc PPase; PSY, phytoene synthase; ZDS, zeta-carotene desaturase; PHT4;2, phosphate transporter; CCD, carotenoid cleavage dioxygenase; PME, pectin methylesterase; 4CL, 4-coumarate:coenzyme A ligase; PE, pectinesterase; PL, pectate lyase; PG, polygalacturonase; PGI, polygalacturonase inhibitor; XET, xyloglucan endotransglucosylase/hydrolase; MANA, α-mannosidase; ZEP, zeaxanthin epoxidase; NCED, 9-cis-epoxy-carotenoid dioxygenase; PYL, PYR1-like; ACO, ACC oxidase; ETR, ethylene receptor; ERF, ethylene responsive factor; SRSF, serine/arginine-rich splicing factor; PSMD, 26S proteasome non-ATPase regulatory subunit.

Sugar content is determined by both phloem unloading and metabolism in the flesh of cultivated watermelon (Guo et al., 2015). The main sugars of cucurbit plants are stachyose, sucrose and raffinose, which are transported from leaves to fruits in the phloem (Mitchell et al., 1992; Chen et al., 2001). α*-galactosidase 2* (*AGA2*), *vacuolar sugar transporter 1* (*VST1*), *tonoplast sugar transporter 2* (*TST2*), *trehalose-phosphate synthase* (*TPS*), *sucrose phosphate synthase* (*SPS*), *sucrose synthase* (*SUS*), *insoluble acid invertase* (*IAI*), *raffinose synthase* (*RFS*), and *UDP-Gal/Glc PPase* (*UGGP*) were reported to be involved in sugar metabolism and accumulation during fruit ripening (Eastmond et al., 2002; Jia et al., 2013a; Ren et al., 2018, 2020, 2021; Durán-Soria et al., 2020). Our RNA-seq and proteomic results showed that most of these genes were consistently expressed at mRNA and protein levels during fruit ripening except for *AGA2* and *TPS* (*Cla97C01G023840*) (Figure 6 and Supplementary Datas 1, 2).

Lycopene predominantly accumulates in the red flesh of watermelon (Holden et al., 1999). The contents of lycopene and β-carotene gradually increase during fruit ripening in cultivated watermelon (Guo et al., 2015). *Phytoene synthase* (*PSY*), *9-cis-epoxy-carotenoid dioxygenase* (*NCED*), *Zeta-carotene desaturase* (*ZDS*), *Carotenoid cleavage dioxygenase* (*CCD*), and *ClPHT4;2* were reported to play crucial roles in carotenoid biosynthesis (Zhang et al., 2017; Sun et al., 2018; McQuinn et al., 2020). These five genes exhibited an overall up-regulated expression pattern at both mRNA and protein levels (Figure 6 and Supplementary Datas 1, 2).

The fruit primary cell wall includes pectin, hemicellulose, cellulose and some structural proteins (Giovannoni, 2001). Fruit softening and textural changes are mainly caused by cell wall depolymerization and solubilization (Giovannoni, 2001; Guo et al., 2015). *Pectin methylesterase* (*PME*), *4-coumarate:coenzyme A ligase* (*4CL*), *pectinesterase* (*PE*), *polygalacturonase* (*PG*), *polygalacturonase inhibitor* (*PGI*), *pectate lyase* (*PL*), *xyloglucan endotransglucosylase/hydrolase* (*XET*), and α*-mannosidase* (*MANA*) have been widely reported to participate in fruit softening (Giovannoni, 2001; Yun et al., 2019). We found that most of these genes were highly expressed at 10 DAP, and gradually decreased at both mRNA and protein levels during fruit ripening (Figure 6 and Supplementary Datas 1, 2). Consistently, down-regulated cell wall pathway was enriched by GO enrichment analysis in both18 DAP vs 10 DAP group and 26 DAP vs 18 DAP group.

Abscisic acid and ethylene have been regarded as the ripening hormones in non-climacteric and climacteric fruits, respectively (Jia et al., 2011; Chen et al., 2020). However, increasing evidence indicates that ethylene is also involved in regulation of non-climacteric fruits ripening, such as strawberry and citrus (Katz et al., 2004; Trainotti et al., 2005; Iannetta et al., 2006). Our proteomic results showed that two ABA biosynthesis-related genes [*zeaxanthin epoxidase* (*ZEP*) and *9-cis-epoxy-carotenoid dioxygenase* (*NCED*)] and two ABA receptors [*PYR1-like 8* (*PYL8*) and *PYL9*] exhibited an upregulation pattern at both mRNA and protein levels (Figure 6). In addition, we found that one ethylene biosynthesis-related gene *ACC oxidase* (*ACO*, *Cla97C04G079350*), two *ethylene receptors* (*ETRs*) and one *ethylene responsive factor* (*ERF*, *Cla97C05G098050*) displayed up-regulated expression pattern, while two *ACOs* (*Cla97C02G043890* and *Cla97C09G173910*) were down-regulated, and one *ERF* (*Cla97C06G109360*) showed an inconsistent expression pattern at the mRNA and protein levels during fruit ripening (Figure 6).

Post-transcriptional and post-translational regulations play important roles in gene expression (Kwak et al., 2011; Robles and Quesada, 2019). Post-transcriptional regulation is commonly mediated by many factors, such as RNA-binding proteins (RBPs), small RNAs, and spliceosome (Xue et al., 2021). Serine/arginine-rich (SR) proteins are important parts of the spliceosome and play key roles in alternative splicing (AS) (Sperling, 2019). We found that several SR proteins and other components of spliceosome changed significantly at the protein level during fruit ripening (Figure 6 and Supplementary Table 2 and Supplementary Data 5). Consistently, the spliceosome was enriched by KEGG pathway enrichment analysis in 18 DAP vs 10 DAP group (Figure 2G and Supplementary Data 5). Ubiquitin-Proteasome System (UPS) is usually known as a protein-degrading machine at the post-translational level. In addition, it also regulates gene transcription at many stages, such as regulation of transcriptional elongation and recruitment of activators to the promoter region of genes (Kwak et al., 2011). Our proteomic results showed that UPS-related proteins were differentially expressed during ripening, such as E3 ubiquitin ligases and 26S proteasome non-ATPase regulatory subunit (PSMD) (Figure 6 and Supplementary Tables 3, 4 and Supplementary Datas 2, 4, 9). Consistently, the up-regulated proteasome pathway was significantly enriched by GO and KEGG pathway enrichment analysis (Figures 2G,H and Supplementary Datas 4, 9).

## Discussion

Watermelon is an important fruit crop and becomes an ideal model plant for studies on non-climacteric fruit ripening. However, the molecular mechanisms of non-climacteric fruit ripening remain largely unknown. Using the high-throughput RNA-seq and quantitative proteome technology, we quantified 18,856 mRNAs and 6,226 proteins from four key fruit development stages in watermelon. Our comparative analysis of the transcriptome and proteome provides a better understanding of the complex regulatory networks of fruit ripening in watermelon.

Gene Ontology enrichment analysis of down-regulated proteins in three groups showed that down-regulated cell wall process was significantly enriched in 18 DAP vs 10 DAP and 26 DAP vs 18 DAP groups but not 34 DAP vs 26 DAP group (Figure 2H and Supplementary Datas 4, 6, 8). This is consistent with the softening process of watermelon flesh. Similar results were also reported in citrus and tomato (Giovannoni, 2004; Wang et al., 2017a), they found that the pectinesterases were highly expressed prior to fruit maturation, and were gradually down-regulated during fruit ripening. These results suggested that fruit softening-related proteins played crucial roles in the early stages of fruit ripening. KEGG pathway enrichment analysis of up-regulated proteins in three groups revealed that the up-regulated starch and sucrose metabolism pathway was enriched in 18 DAP vs 10 DAP and 34 DAP vs 26 DAP groups (Supplementary Datas 5, 9). The up-regulation of starch and sucrose metabolism was consistent with the increase of sugar content during fruit ripening (Figure 1E). Phenolic compounds are produced by the phenylpropanoid pathway and contribute to fruit pigmentation and the disease resistance response in many fleshy fruits (Singh et al., 2010). Consistent with the previous study in pear (Li et al., 2015), our KEGG pathway enrichment analysis also demonstrated that phenylpropanoid biosynthesis was enriched at the early developmental stages of fruit ripening (Figure 2G and Supplementary Data 5).

Abscisic acid is a major endogenous factor and the primary signal that regulates the ripening of non-climacteric fruits (Jia et al., 2011; Leng et al., 2014; Wang et al., 2017b; Fenn and Giovannoni, 2021; Kou et al., 2021). Previous studies also reported that ABA played a direct or indirect role in the induction of phenylpropanoid biosynthesis during fruit ripening (Jaakola, 2013; Vighi et al., 2019). We found that two ABA biosynthesis-related genes (*ZEP* and *NCED*) and two ABA receptors (*PYL8* and *PYL9*) displayed an up-regulated expression pattern at both mRNA and protein levels during ripening (Figure 6 and Supplementary Data 2), suggesting that ABA signal transduction was enhanced during fruit ripening. The enhanced ABA signaling might account for up-regulated phenylpropanoid biosynthesis at the early stages of watermelon fruit ripening (Supplementary Data 5). Additionally, *NCED* and *PYL9* were reported to be involved in regulation of tomato fruit ripening (Sun et al., 2012; Kai et al., 2019). Taken together, our and previous studies indicated that ABA might play key roles in fruit ripening, especially for non-climacteric fruits. Ethylene has long been known to play a major role in the ripening process of climacteric fruits (Klee, 2002; Chen et al., 2020; Fenn and Giovannoni, 2021). Increasing evidences have indicated that ethylene is also involved in regulation of non-climacteric fruits ripening (Chervin et al., 2004; Trainotti et al., 2005). Our proteomic results showed that ethylene biosynthesis-related genes, ethylene receptors and ethylene signaling regulators were differentially expressed during fruit ripening (Figure 6 and Supplementary Data 2), implying possible roles of ethylene in regulation of watermelon fruit ripening.

Fruit set initiation is modulated by the combined accumulation of auxin and GA in many fleshy fruits (Srivastava and Handa, 2005; Fenn and Giovannoni, 2021). In addition, auxin signaling and auxin-GA interaction are key elements influencing fruit growth, such as regulation of cell division and expansion during fruit ripening (Fenn and Giovannoni, 2021). Our GO enrichment analysis showed that auxin and GA biosynthesis/metabolism-related proteins were significantly up-regulated in 18 DAP vs 10 DAP (Supplementary Table 5 and Supplementary Data 4), implying these proteins might play important roles in early stage of watermelon fruit development. Future work will study the potential mechanisms of these proteins in regulation of fruit development, especially whether these proteins affect fruit size.

Base on our proteome methodology, integrative analysis of the proteome and transcriptome showed that smaller changes occurred in protein abundances than in mRNA abundances during fruit ripening (Supplementary Datas 1, 2, 11), and the correlation coefficients (*R*^2^) decreased during fruit ripening, indicating that the relationship between mRNAs and proteins weakened during the ripening process (Figure 4B). These results were consistent with studies in tomato (Belouah et al., 2019). Taken together, these consistent and solid results in watermelon and tomato indicated that post-transcriptional and post-translational mechanisms might play important roles in the regulation of fruit-related genes expression. Our proteomic results showed that alternative splicing-associated proteins were differentially expressed during fruit ripening, such as SR proteins and RNPs (Figure 6 and Supplementary Datas 2, 5). Furthermore, KEGG pathway enrichment analysis showed that spliceosome-related proteins were down-regulated in 18 DAP vs 10 DAP group, but up-regulated in 26 DAP vs 18 DAP group (Supplementary Table 2 and Supplementary Datas 5, 7). The dynamic expression of these alternative splicing-associated and spliceosome-related proteins indicated that abundant post-transcriptional regulatory events occurred during fruit ripening. Our proteomic results also showed that there were more down-regulated proteins than up-regulated proteins in three groups, and more proteins were down-regulated in 18 DAP vs 10 DAP and 34 DAP vs 26 DAP groups (Figure 2D and Supplementary Data 2). Consistently, GO and KEGG pathway enrichment analysesshowed that proteasome-associated and ubiquitin mediated proteolysis-related proteins were significantly up-regulated in 18 DAP vs 10 DAP and 34 DAP vs 26 DAP groups but not 26 DAP vs 18 DAP group (Supplementary Tables 3, 4 and Supplementary Datas 4, 9), implying that these down-regulated proteins in 18 DAP vs 10 DAP and 34 DAP vs 26 DAP groups might be regulated by proteasomal degradation at the post-translational level, especially for those down-regulated proteins whose expression is not down-regulated at the mRNA level (Supplementary Figure 2). Interestingly, GSEA showed that both spliceosome and proteasome displayed a negative correlation during ripening, indicating that these proteins themselves might be regulated by post-transcriptional and post-translational mechanisms (Figure 4C). Additionally, the weak correlation of spliceosome and proteasome at the mRNA and protein levels was also confirmed by hierarchical clustering analysis (Figure 5). Taken together, the results of our GO and KEGG pathway enrichment analyses, hierarchical clustering analysis and comparative analysis between mRNAs and proteins suggested that post-transcriptional and post-translational mechanisms might play important roles in fruit ripening by regulating the expression of fruit ripening-related genes (Figures 2G, 4B,C, 5 and Supplementary Tables 2–4 and Supplementary Datas 4, 5, 7, 9). These post-transcriptional and post-translational regulations might account for the poor correlation between mRNAs and proteins during ripening. Indeed, we found that at least 6,266 genes underwent AS during watermelon fruit ripening (data not shown). Most recently, MaMYB16L was reported to regulate starch degradation by alternative splicing in banana fruit during ripening (Jiang et al., 2021). These results support the potential post-transcriptional and post-translational mechanisms in regulation of fruit ripening, which have also been described in other fruits, such as tomato, sweet orange and citrus (Osorio et al., 2011; Pan et al., 2012; Wu et al., 2014b; Belouah et al., 2019). We will focus on post-transcriptional and post-translational regulation of watermelon development and ripening in future work.

## Data Availability Statement

Our RNA-seq data were submitted to the SRA of NCBI under the accession number PRJNA718123. Proteomic data were uploaded to the Pride_EBI database (http://www.ebi.ac.uk/pride), and are available via ProteomeXchange with identifier PXD024490.

## Author Contributions

YY and YX conceived and designed the study and wrote the manuscript with contributions from all co-authors. SG and HS provided good advice for data analysis. YR, JZ, and ML collected and grew the plant material. JW, YZ, YC, GG, and HZ provided critical comments and edited the manuscript. ST revised the manuscript and gave good advice for the revised manuscript. All authors approved the manuscript.

## Conflict of Interest

The authors declare that the research was conducted in the absence of any commercial or financial relationships that could be construed as a potential conflict of interest.

## Publisher’s Note

All claims expressed in this article are solely those of the authors and do not necessarily represent those of their affiliated organizations, or those of the publisher, the editors and the reviewers. Any product that may be evaluated in this article, or claim that may be made by its manufacturer, is not guaranteed or endorsed by the publisher.

## Footnotes

1
http://cucurbitgenomics.org/ftp/genome/watermelon/97103/v2/

2
https://www.ebi.ac.uk/GOA/

## References

[B1] Adams-Phillips L. Barry C. Giovannoni J. (2004). Signal transduction systems regulating fruit ripening. *Trends Plant Sci.* 9 331–338. 10.1016/j.tplants.2004.05.004 15231278

[B2] Alexander L. Grierson D. (2002). Ethylene biosynthesis and action in tomato: a model for climacteric fruit ripening. *J. Exp. Bot.* 53 2039–2055. 10.1093/jxb/erf072 12324528

[B3] Anders S. Huber W. (2010). Differential expression analysis for sequence count data. *Genome Biol.* 11:R106. 10.1186/gb-2010-11-10-r106 20979621 PMC3218662

[B4] Anders S. Pyl P. T. Huber W. (2015). HTSeq–a Python framework to work with high-throughput sequencing data. *Bioinformatics* 31 166–169. 10.1093/bioinformatics/btu638 25260700 PMC4287950

[B5] Belouah I. Nazaret C. Pétriacq P. Prigent S. Bénard C. Mengin V. (2019). Modeling protein destiny in developing fruit. *Plant Physiol.* 180 1709–1724. 10.1104/pp.19.00086 31015299 PMC6752906

[B6] Benjamini Y. Hochberg Y. (1995). Controlling the false discovery rate: a practical and powerful approach to multiple testing. *J. R.Stat. Soc. Ser. B.* 57 289–300. 10.1111/j.2517-6161.1995.tb02031.x

[B7] Branham S. Vexler L. Meir A. Tzuri G. Frieman Z. Levi A. (2017). Genetic mapping of a major codominant QTL associated with β-carotene accumulation in watermelon. *Mol. Breed.* 37:146. 10.1007/s11032-017-0747-0

[B8] Chai Y. M. Jia H. F. Li C. L. Dong Q. H. Shen Y. Y. (2011). FaPYR1 is involved in strawberry fruit ripening. *J. Exp. Bot.* 62 5079–5089. 10.1093/jxb/err207 21778181

[B9] Chen S. Petersen B. L. Olsen C. E. Schulz A. Halkier B. A. (2001). Long-distance phloem transport of glucosinolates in Arabidopsis. *Plant Physiol.* 127 194–201. 10.1104/pp.127.1.194 11553747 PMC117975

[B10] Chen T. Qin G. Tian S. (2020). Regulatory network of fruit ripening: current understanding and future challenges. *New Phytol.* 228 1219–1226. 10.1111/nph.16822 32729147

[B11] Chervin C. El-Kereamy A. Roustan J.-P. Latché A. Lamon J. Bouzayen M. (2004). Ethylene seems required for the berry development and ripening in grape, a non-climacteric fruit. *Plant Sci.* 167 1301–1305. 10.1016/j.plantsci.2004.06.026

[B12] Durán-Soria S. Pott D. M. Osorio S. Vallarino J. G. (2020). Sugar signaling during fruit ripening. *Front. Plant Sci.* 11:564917. 10.3389/fpls.2020.564917 32983216 PMC7485278

[B13] Eastmond P. J. van Dijken A. J. Spielman M. Kerr A. Tissier A. F. Dickinson H. G. (2002). Trehalose-6-phosphate synthase 1, which catalyses the first step in trehalose synthesis, is essential for Arabidopsis embryo maturation. *Plant J.* 29 225–235. 10.1046/j.1365-313x.2002.01220.x 11851922

[B14] Erickson D. L. Smith B. D. Clarke A. C. Sandweiss D. H. Tuross N. (2005). An Asian origin for a 10,000-year-old domesticated plant in the Americas. *Proc. Natl. Acad. Sci. U.S.A.* 102 18315–18320. 10.1073/pnas.0509279102 16352716 PMC1311910

[B15] Fenn M. A. Giovannoni J. J. (2021). Phytohormones in fruit development and maturation. *Plant J.* 105 446–458. 10.1111/tpj.15112 33274492

[B16] Gibon Y. Usadel B. Blaesing O. E. Kamlage B. Hoehne M. Trethewey R. (2006). Integration of metabolite with transcript and enzyme activity profiling during diurnal cycles in Arabidopsis rosettes. *Genome Biol.* 7:R76. 10.1186/gb-2006-7-8-R76 16916443 PMC1779593

[B17] Giovannoni J. (2001). Molecular biology of fruit maturation and ripening. *Annu. Rev. Plant Physiol. Plant Mol. Biol.* 52 725–749. 10.1146/annurev.arplant.52.1.725 11337414

[B18] Giovannoni J. J. (2004). Genetic regulation of fruit development and ripening. *Plant Cell* 16(Suppl.), S170–S180. 10.1105/tpc.019158 15010516 PMC2643394

[B19] Grassi S. Piro G. Lee J. M. Zheng Y. Fei Z. Dalessandro G. (2013). Comparative genomics reveals candidate carotenoid pathway regulators of ripening watermelon fruit. *BMC Genomics* 14:781. 10.1186/1471-2164-14-781 24219562 PMC3840736

[B20] Guo S. Sun H. Zhang H. Liu J. Ren Y. Gong G. (2015). Comparative Transcriptome Analysis of Cultivated and Wild Watermelon during Fruit Development. *PLoS One* 10:e0130267. 10.1371/journal.pone.0130267 26079257 PMC4469606

[B21] Guo S. Zhang J. Sun H. Salse J. Lucas W. J. Zhang H. (2013). The draft genome of watermelon (*Citrullus lanatus*) and resequencing of 20 diverse accessions. *Nat. Genet.* 45 51–58. 10.1038/ng.2470 23179023

[B22] Guo S. Zhao S. Sun H. Wang X. Wu S. Lin T. (2019). Resequencing of 414 cultivated and wild watermelon accessions identifies selection for fruit quality traits. *Nat. Genet.* 51 1616–1623. 10.1038/s41588-019-0518-4 31676863

[B23] Han Y. Dang R. Li J. Jiang J. Zhang N. Jia M. (2015). Sucrose nonfermenting1-related protein kinase2.6, an ortholog of OPEN STOMATA1, is a negative regulator of strawberry fruit development and ripening. *Plant Physiol.* 167 915–930. 10.1104/pp.114.251314 25609556 PMC4348756

[B24] Harvald E. B. Sprenger R. R. Dall K. B. Ejsing C. S. Nielsen R. Mandrup S. (2017). Multi-omics analyses of starvation responses reveal a central role for lipoprotein metabolism in acute starvation survival in *C. elegans*. *Cell Syst.* 5 .e34–.e52. 10.1016/j.cels.2017.06.004 28734827

[B25] Holden J. M. Eldridge A. L. Beecher G. R. Marilyn Buzzard I. Bhagwat S. Davis C. S. (1999). Carotenoid content of U.S. foods: an update of the database. *J. Food Composition Anal.* 12 169–196. 10.1006/jfca.1999.0827

[B26] Huang K. Zhou S. Shen K. Zhou Y. Wang F. Jiang X. (2020). Elucidation of the miR164c-guided gene/protein interaction network controlling seed vigor in rice. *Front. Plant Sci.* 11:589005. 10.3389/fpls.2020.589005 33281848 PMC7688992

[B27] Iannetta P. P. M. Laarhoven L.-J. Medina-Escobar N. James E. K. McManus M. T. Davies H. V. (2006). Ethylene and carbon dioxide production by developing strawberries show a correlative pattern that is indicative of ripening climacteric fruit. *Physiol. Plant.* 127 247–259. 10.1111/j.1399-3054.2006.00656.x

[B28] Jaakola L. (2013). New insights into the regulation of anthocyanin biosynthesis in fruits. *Trends Plant Sci.* 18 477–483. 10.1016/j.tplants.2013.06.003 23870661

[B29] Jia H. Wang Y. Sun M. Li B. Han Y. Zhao Y. (2013a). Sucrose functions as a signal involved in the regulation of strawberry fruit development and ripening. *New Phytol.* 198 453–465. 10.1111/nph.12176 23425297

[B30] Jia H. F. Lu D. Sun J. H. Li C. L. Xing Y. Qin L. (2013b). Type 2C protein phosphatase ABI1 is a negative regulator of strawberry fruit ripening. *J. Exp. Bot.* 64 1677–1687. 10.1093/jxb/ert028 23404898 PMC3617833

[B31] Jia H. F. Chai Y. M. Li C. L. Lu D. Luo J. J. Qin L. (2011). Abscisic acid plays an important role in the regulation of strawberry fruit ripening. *Plant Physiol.* 157 188–199. 10.1104/pp.111.177311 21734113 PMC3165869

[B32] Jiang G. Zhang D. Li Z. Liang H. Deng R. Su X. (2021). Alternative splicing of MaMYB16L regulates starch degradation in banana fruit during ripening. *J. Integr. Plant Biol.* 63 1341–1352. 10.1111/jipb.13088 33656245

[B33] Kai W. Wang J. Liang B. Fu Y. Zheng Y. Zhang W. (2019). PYL9 is involved in the regulation of ABA signaling during tomato fruit ripening. *J. Exp. Bot.* 70 6305–6319. 10.1093/jxb/erz396 31504753 PMC6859720

[B34] Katz E. Lagunes P. M. Riov J. Weiss D. Goldschmidt E. E. (2004). Molecular and physiological evidence suggests the existence of a system II-like pathway of ethylene production in non-climacteric Citrus fruit. *Planta* 219 243–252. 10.1007/s00425-004-1228-3 15014996

[B35] Klee H. J. (2002). Control of ethylene-mediated processes in tomato at the level of receptors. *J. Exp. Bot.* 53 2057–2063. 10.1093/jxb/erf062 12324529

[B36] Klee H. J. Giovannoni J. J. (2011). Genetics and control of tomato fruit ripening and quality attributes. *Annu. Rev. Genet.* 45 41–59. 10.1146/annurev-genet-110410-132507 22060040

[B37] Kou X. Yang S. Chai L. Wu C. Zhou J. Liu Y. (2021). Abscisic acid and fruit ripening: multifaceted analysis of the effect of abscisic acid on fleshy fruit ripening. *Sci. Hortic.* 281:109999. 10.1016/j.scienta.2021.109999

[B38] Kwak J. Workman J. L. Lee D. (2011). The proteasome and its regulatory roles in gene expression. *Biochim. Biophys. Acta* 1809 88–96. 10.1016/j.bbagrm.2010.08.001 20723625

[B39] Leng P. Yuan B. Guo Y. (2014). The role of abscisic acid in fruit ripening and responses to abiotic stress. *J. Exp. Bot.* 65 4577–4588. 10.1093/jxb/eru204 24821949

[B40] Li C. Jia H. Chai Y. Shen Y. (2011). Abscisic acid perception and signaling transduction in strawberry: a model for non-climacteric fruit ripening. *Plant Signal Behav.* 6 1950–1953. 10.4161/psb.6.12.18024 22095148 PMC3337185

[B41] Li J. M. Huang X. S. Li L. T. Zheng D. M. Xue C. Zhang S. L. (2015). Proteome analysis of pear reveals key genes associated with fruit development and quality. *Planta* 241 1363–1379. 10.1007/s00425-015-2263-y 25682102

[B42] Liu M. Pirrello J. Chervin C. Roustan J. P. Bouzayen M. (2015). Ethylene control of fruit ripening: revisiting the complex network of transcriptional regulation. *Plant Physiol.* 169 2380–2390. 10.1104/pp.15.01361 26511917 PMC4677914

[B43] Manning K. Tör M. Poole M. Hong Y. Thompson A. J. King G. J. (2006). A naturally occurring epigenetic mutation in a gene encoding an SBP-box transcription factor inhibits tomato fruit ripening. *Nat. Genet.* 38 948–952. 10.1038/ng1841 16832354

[B44] McQuinn R. P. Gapper N. E. Gray A. G. Zhong S. Tohge T. Fei Z. (2020). Manipulation of ZDS in tomato exposes carotenoid- and ABA-specific effects on fruit development and ripening. *Plant Biotechnol. J.* 18 2210–2224. 10.1111/pbi.13377 32171044 PMC7589306

[B45] Mertins P. Mani D. R. Ruggles K. V. Gillette M. A. Clauser K. R. Wang P. (2016). Proteogenomics connects somatic mutations to signalling in breast cancer. *Nature* 534 55–62. 10.1038/nature18003 27251275 PMC5102256

[B46] Mitchell D. E. Gadus M. V. Madore M. A. (1992). Patterns of assimilate production and translocation in muskmelon (*Cucumis melo* L.) : I. diurnal patterns. *Plant Physiol.* 99 959–965. 10.1104/pp.99.3.959 16669025 PMC1080570

[B47] Murtagh F. Legendre P. (2014). Ward’s hierarchical agglomerative clustering method: which algorithms implement ward’s criterion? *J. Classif.* 31 274–295. 10.1007/s00357-014-9161-z

[B48] Osorio S. Alba R. Damasceno C. M. Lopez-Casado G. Lohse M. Zanor M. I. (2011). Systems biology of tomato fruit development: combined transcript, protein, and metabolite analysis of tomato transcription factor (nor, rin) and ethylene receptor (Nr) mutants reveals novel regulatory interactions. *Plant Physiol.* 157 405–425. 10.1104/pp.111.175463 21795583 PMC3165888

[B49] Palma J. M. Corpas F. J. del Río L. A. (2011). Proteomics as an approach to the understanding of the molecular physiology of fruit development and ripening. *J. Proteomics* 74 1230–1243. 10.1016/j.jprot.2011.04.010 21524723

[B50] Pan Z. Zeng Y. An J. Ye J. Xu Q. Deng X. (2012). An integrative analysis of transcriptome and proteome provides new insights into carotenoid biosynthesis and regulation in sweet orange fruits. *J. Proteomics* 75 2670–2684. 10.1016/j.jprot.2012.03.016 22472342

[B51] Pech J.-C. Purgatto E. Bouzayen M. Latché A. (2012). Ethylene and fruit ripening. *Annu. Plant Rev.* 44 275–304.

[B52] Quinet M. Angosto T. Yuste-Lisbona F. J. Blanchard-Gros R. Bigot S. Martinez J. P. (2019). Tomato fruit development and metabolism. *Front. Plant Sci.* 10:1554. 10.3389/fpls.2019.01554 31850035 PMC6895250

[B53] Ren Y. Guo S. Zhang J. He H. Sun H. Tian S. (2018). A tonoplast sugar transporter underlies a sugar accumulation QTL in watermelon. *Plant Physiol.* 176 836–850. 10.1104/pp.17.01290 29118248 PMC5761790

[B54] Ren Y. Li M. Guo S. Sun H. Zhao J. Zhang J. (2021). Evolutionary gain of oligosaccharide hydrolysis and sugar transport enhanced carbohydrate partitioning in sweet watermelon fruits. *Plant Cell* 33 1554–1573. 10.1093/plcell/koab055 33570606 PMC8254481

[B55] Ren Y. Sun H. Zong M. Guo S. Ren Z. Zhao J. (2020). Localization shift of a sugar transporter contributes to phloem unloading in sweet watermelons. *New Phytol.* 227 1858–1871. 10.1111/nph.16659 32453446

[B56] Robles P. Quesada V. (2019). Transcriptional and post-transcriptional regulation of organellar gene expression (oge) and its roles in plant salt tolerance. *Int. J. Mol. Sci.* 20:1056. 10.3390/ijms20051056 30823472 PMC6429081

[B57] Seymour G. B. Østergaard L. Chapman N. H. Knapp S. Martin C. (2013). Fruit development and ripening. *Annu. Rev. Plant Biol.* 64 219–241. 10.1146/annurev-arplant-050312-120057 23394500

[B58] Singh R. Rastogi S. Dwivedi U. N. (2010). phenylpropanoid metabolism in ripening fruits. *Compr. Rev. Food Sci. Food Saf.* 9 398–416. 10.1111/j.1541-4337.2010.00116.x 33467837

[B59] Sperling R. (2019). Small non-coding RNA within the endogenous spliceosome and alternative splicing regulation. *Biochim. Biophys. Acta Gene Regul. Mech.* 1862:194406. 10.1016/j.bbagrm.2019.07.007 31323432

[B60] Srivastava A. Handa A. K. (2005). Hormonal regulation of tomato fruit development: a molecular perspective. *J. Plant Growth Regul.* 24 67–82. 10.1007/s00344-005-0015-0

[B61] Subramanian A. Tamayo P. Mootha V. K. Mukherjee S. Ebert B. L. Gillette M. A. (2005). Gene set enrichment analysis: a knowledge-based approach for interpreting genome-wide expression profiles. *Proc. Natl. Acad. Sci. U.S.A.* 102 15545–15550. 10.1073/pnas.0506580102 16199517 PMC1239896

[B62] Sun L. Sun Y. Zhang M. Wang L. Ren J. Cui M. (2012). Suppression of 9-cis-epoxycarotenoid dioxygenase, which encodes a key enzyme in abscisic acid biosynthesis, alters fruit texture in transgenic tomato. *Plant Physiol.* 158 283–298. 10.1104/pp.111.186866 22108525 PMC3252109

[B63] Sun T. Yuan H. Cao H. Yazdani M. Tadmor Y. Li L. (2018). Carotenoid metabolism in plants: the role of plastids. *Mol. Plant* 11 58–74. 10.1016/j.molp.2017.09.010 28958604

[B64] Trainotti L. Pavanello A. Casadoro G. (2005). Different ethylene receptors show an increased expression during the ripening of strawberries: does such an increment imply a role for ethylene in the ripening of these non-climacteric fruits? *J. Exp. Bot.* 56 2037–2046. 10.1093/jxb/eri202 15955790

[B65] Trapnell C. Pachter L. Salzberg S. L. (2009). TopHat: discovering splice junctions with RNA-Seq. *Bioinformatics* 25 1105–1111. 10.1093/bioinformatics/btp120 19289445 PMC2672628

[B66] Umer M. J. Bin Safdar L. Gebremeskel H. Zhao S. Yuan P. Zhu H. (2020). Identification of key gene networks controlling organic acid and sugar metabolism during watermelon fruit development by integrating metabolic phenotypes and gene expression profiles. *Hortic. Res.* 7:193. 10.1038/s41438-020-00416-8 33328462 PMC7705761

[B67] Vighi I. L. Crizel R. L. Perin E. C. Rombaldi C. V. Galli V. (2019). Crosstalk During Fruit Ripening and Stress Response Among Abscisic Acid, Calcium-Dependent Protein Kinase and Phenylpropanoid. *Crit. Rev. Plant Sci.* 38 99–116. 10.1080/07352689.2019.1602959

[B68] Vrebalov J. Ruezinsky D. Padmanabhan V. White R. Medrano D. Drake R. (2002). A MADS-box gene necessary for fruit ripening at the tomato ripening-inhibitor (rin) locus. *Science* 296 343–346. 10.1126/science.1068181 11951045

[B69] Wang J. H. Liu J. J. Chen K. L. Li H. W. He J. Guan B. (2017a). Comparative transcriptome and proteome profiling of two Citrus sinensis cultivars during fruit development and ripening. *BMC Genomics* 18:984. 10.1186/s12864-017-4366-2 29268697 PMC5740884

[B70] Wang Y. Guo S. Tian S. Zhang J. Ren Y. Sun H. (2017b). Abscisic acid pathway involved in the regulation of watermelon fruit ripening and quality trait evolution. *PLoS One* 12:e0179944. 10.1371/journal.pone.0179944 28662086 PMC5491074

[B71] Wang Z. Zhang R. Liu F. Jiang P. Xu J. Cao H. (2019). TMT-based quantitative proteomic analysis reveals proteomic changes involved in longevity. *Proteomics Clin. Appl.* 13:e1800024. 10.1002/prca.201800024 30485681

[B72] Wienkoop S. Morgenthal K. Wolschin F. Scholz M. Selbig J. Weckwerth W. (2008). Integration of metabolomic and proteomic phenotypes: analysis of data covariance dissects starch and RFO metabolism from low and high temperature compensation response in Arabidopsis thaliana. *Mol. Cell Proteomics* 7 1725–1736. 10.1074/mcp.M700273-MCP200 18445580 PMC2556022

[B73] Wu H. X. Jia H. M. Ma X. W. Wang S. B. Yao Q. S. Xu W. T. (2014a). Transcriptome and proteomic analysis of mango (*Mangifera indica* Linn) fruits. *J. Proteomics* 105 19–30. 10.1016/j.jprot.2014.03.030 24704857

[B74] Wu J. Xu Z. Zhang Y. Chai L. Yi H. Deng X. (2014b). An integrative analysis of the transcriptome and proteome of the pulp of a spontaneous late-ripening sweet orange mutant and its wild type improves our understanding of fruit ripening in citrus. *J. Exp. Bot.* 65 1651–1671. 10.1093/jxb/eru044 24600016 PMC3967095

[B75] Xue X. Jiao F. Xu H. Jiao Q. Zhang X. Zhang Y. (2021). The role of RNA-binding protein, microRNA and alternative splicing in seed germination: a field need to be discovered. *BMC Plant Biol.* 21:194. 10.1186/s12870-021-02966-y 33882821 PMC8061022

[B76] Yuan X. Y. Wang R. H. Zhao X. D. Luo Y. B. Fu D. Q. (2016). Role of the tomato non-ripening mutation in regulating fruit quality elucidated using iTRAQ protein profile analysis. *PLoS One* 11:e0164335. 10.1371/journal.pone.0164335 27732677 PMC5061430

[B77] Yue P. Lu Q. Liu Z. Lv T. Li X. Bu H. (2020). Auxin-activated MdARF5 induces the expression of ethylene biosynthetic genes to initiate apple fruit ripening. *New Phytol.* 226 1781–1795. 10.1111/nph.16500 32083754 PMC7317826

[B78] Yun Z. Li T. Gao H. Zhu H. Gupta V. K. Jiang Y. (2019). Integrated transcriptomic, proteomic, and metabolomics analysis reveals peel ripening of harvested banana under natural condition. *Biomolecules* 9:167. 10.3390/biom9050167 31052343 PMC6572190

[B79] Zhang J. Guo S. Ren Y. Zhang H. Gong G. Zhou M. (2017). High-level expression of a novel chromoplast phosphate transporter ClPHT4;2 is required for flesh color development in watermelon. *New Phytol.* 213 1208–1221. 10.1111/nph.14257 27787901

