# Supplementary material for: Quantitative Transcriptomic and Proteomic Analysis of Fruit Development and Ripening in Watermelon (Citrullus lanatus)
Source: Front Plant Sci. 2022 Mar 22;13:818392. doi: 10.3389/fpls.2022.818392 (PMC8980866; doi:10.3389/fpls.2022.818392)
Supplement: Supplementary file 1 [file Data_Sheet_1.docx]

**Supplemental Information**


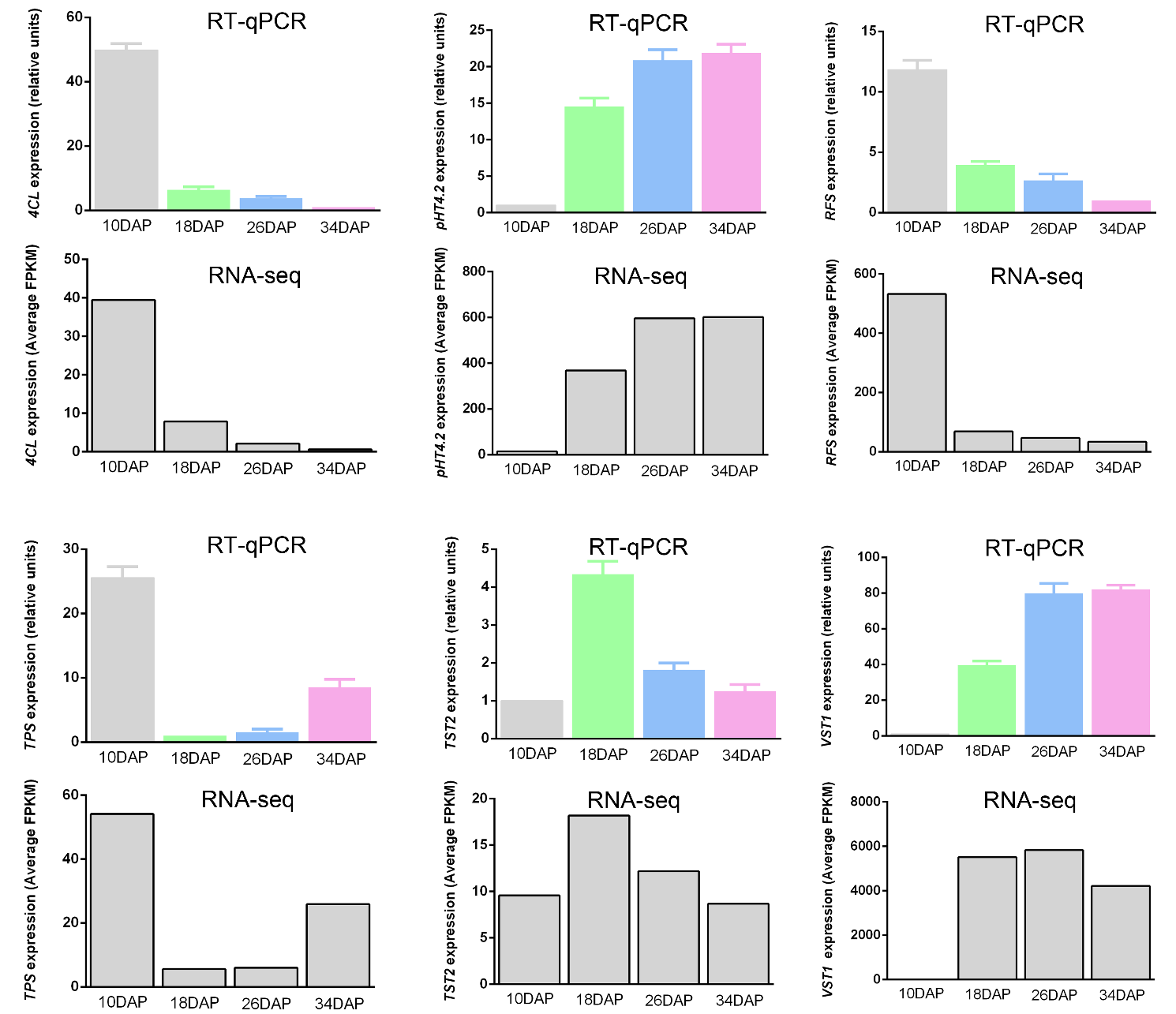


**Supplementary Figure S1**. **Validation of the RNA-seq data by RT-qPCR.**

The watermelon *ACTIN7* gene was used as an internal control. Error bars represent mean ± standard deviation (SD). *4CL(Cla97C05G096310)*: 4-coumarate:coenzyme A ligase; *PHT4;2(Cla97C10G205070)*: phosphate transporter; *RFS (Cla97C06G120620)*: raffinose synthase; *TPS (Cla97C11G223240)*: trehalose-phosphate synthase; *TST2 (Cla97C00G000440)*, tonoplast sugar transporter 2; *VST1 (Cla97C02G031010)*: vacuolar sugar transporter 1;

**
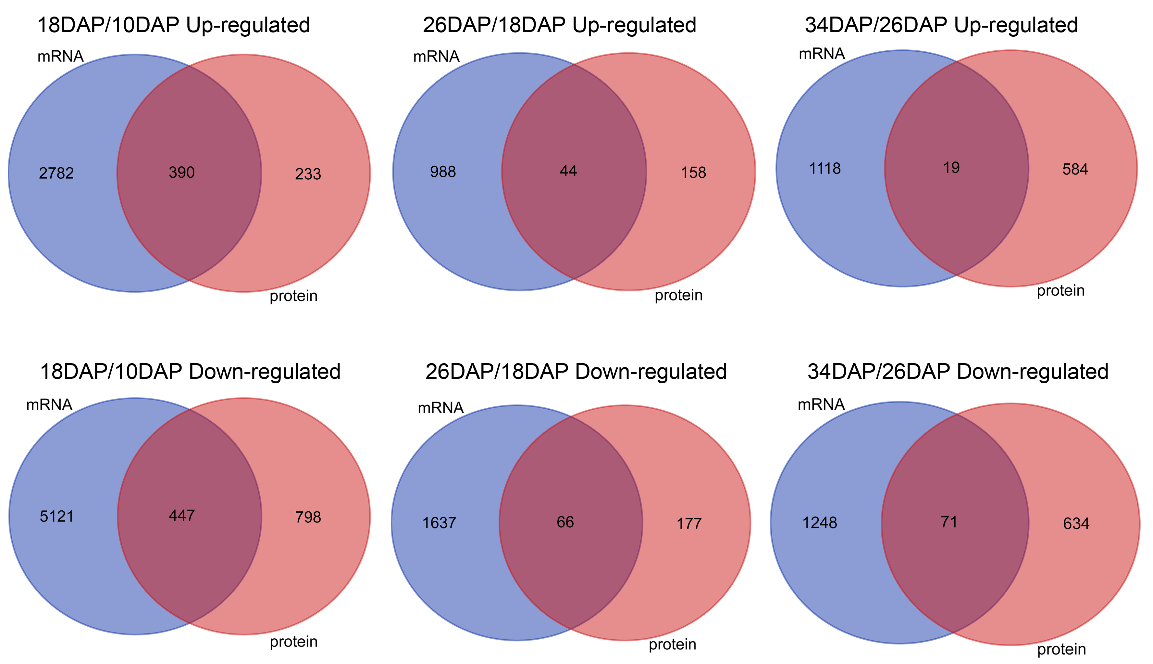
**

**Supplementary Figure S2. Venn diagrams of DEGs and DEPs.**

Co-upregulated and co-downregulated genes and proteins of three groups were showed by venn diagrams.

**Supplemental Table 1.** Primers used for qPCR.

| *Cla97C02G026960* (*ACTIN 7*)  forward primer 5’- CCTACAACTCAATTATGAAGTGTG -3’  reverse primer 5’- GAAATCCACATCTGCTGGAAGGTG-3’ |
| --- |
| *Cla97C00G000440* (*TST2*)  forward primer 5’- TGACCAAGATCTATTGACTGACAAA -3’  reverse primer 5’- AAGAGAGTGACAAGAGGATCGATAA -3’ |
| *Cla97C10G205070* (*PHT4;2*)  forward primer 5’- CTATTATAGTCGCCAATATGAC -3’  reverse primer 5’- GAGTTTGAGATTCCATGGAGAAAG -3’ |
| *Cla97C02G031010* (*VST1*)  forward primer 5’- TTCTCCACCTCCCTCCTGCTCCT -3’  reverse primer 5’- TATACACATCTGCCAGTAAGGGAGTGG -3’ |
| *Cla97C06G120620* (*RFS*)  forward primer 5’- CCGTTCGACGCAATCTCCGATGCGAT -3’  reverse primer 5’- CGGCTGCTTCGGTTGTTTCTCGCC -3’ |
| *Cla97C05G096310* (*4CL*)  forward primer 5’- GTTGAGAAATATCCGAATGTCGCAA -3’  reverse primer 5’- GGCCTCCGTGTTACCAAAGTACC -3’ |
| *Cla97C11G223240* (*TPS*)  forward primer 5’- ATGCCTGGGAATAAGTATAACGGT -3’  reverse primer 5’- CTTGCCATCTTGCTTGTCACATC -3’ |

**Supplemental Table 2.** Up-regulated spliceosome-related proteins in 26 DAP vs 18 DAP.

| **Protein accession** | **Protein description** | **26DAP/18DAP**  **Ratio** | **26DAP/18DAP**  **P value** |
| --- | --- | --- | --- |
| Cla97C03G062650 | Serine/arginine-rich splicing factor SC35 | 1.545 | 0.000120092 |
| Cla97C03G067680 | Arginine/serine-rich splicing factor, putative | 1.702 | 0.000204094 |
| Cla97C05G093540 | THO complex subunit 4D | 1.636 | 0.000219048 |
| Cla97C07G136230 | apoptotic chromatin condensation inducer in the nucleus | 1.514 | 0.000082 |
| Cla97C09G168380 | Arginine/serine-rich splicing factor, putative | 1.458 | 0.000283377 |
| Cla97C10G200740 | Serine/arginine-rich splicing factor SC35 | 1.529 | 0.007028311 |
| Cla97C11G217390 | Heat shock 70 kDa protein | 1.472 | 0.00000619 |
| Cla97C01G006990 | THO complex subunit 2 | 1.391 | 0.047312006 |

**Supplemental Table 3.** Up-regulated ubiquitin mediated proteolysis-related proteins in 18 DAP vs 10 DAP.

| **Protein accession** | **Protein description** | **18DAP/10DAP Ratio** | **18DAP/10DAP P value** |
| --- | --- | --- | --- |
| Cla97C01G000040 | NPL4-like protein 1 | 1.427 | 0.0000677 |
| Cla97C02G028500 | Ubiquitin fusion degradation 1 | 1.46 | 0.000231261 |
| Cla97C02G033150 | Cysteine protease | 1.322 | 0.001543173 |
| Cla97C02G039330 | macrophage erythroblast attacher | 1.339 | 0.001563774 |
| Cla97C02G039830 | E3 ubiquitin ligase BIG BROTHER-related | 1.658 | 0.006226708 |
| Cla97C05G102870 | SKP1-like protein 12 | 1.304 | 0.015202842 |
| Cla97C06G125620 | DNAJ heat shock N-terminal domain-containing protein, putative | 1.345 | 0.013789169 |
| Cla97C07G132430 | coronatine-insensitive protein 1 | 1.47 | 0.000241111 |
| Cla97C10G185830 | 26S proteasome non-ATPase regulatory subunit 13 homolog A | 1.337 | 0.000423441 |
| Cla97C10G192310 | E3 ubiquitin protein ligase | 1.334 | 0.004753204 |

**Supplemental Table 4.** Up-regulated ubiquitin mediated proteolysis-related proteins in 34 DAP vs 26 DAP.

| **Protein accession** | **Protein description** | **34DAP/26DAP Ratio** | **34DAP/26DAP P value** |
| --- | --- | --- | --- |
| Cla97C02G045340 | ubiquitin-activating enzyme E1 | 1.488 | 0.00000135 |
| Cla97C03G058090 | cullin-4 | 2.104 | 0.0000104 |
| Cla97C05G102870 | SKP1-like protein 12 | 2.096 | 0.000146688 |
| Cla97C06G114750 | cullin-1 | 1.489 | 0.001554141 |
| Cla97C08G154570 | E3 ubiquitin-protein ligase UPL1-like | 1.731 | 0.001027123 |
| Cla97C10G188840 | E3 ubiquitin-protein ligase UPL1 | 1.543 | 0.000158207 |
| Cla97C10G197840 | cullin-3A-like | 1.573 | 0.00537066 |
| Cla97C10G198280 | Ubiquitin conjugation factor E4, putative | 1.519 | 0.00102536 |

**Supplemental Table 5.** Up-regulated auxin and GA biosynthesis/metabolism-related proteins in 18 DAP vs 10 DAP.

| **Protein accession** | **Protein description** | **18DAP/10DAP Ratio** | **18DAP/10DAP P value** |
| --- | --- | --- | --- |
| Cla97C01G010050.1 | tropinone reductase-like 3 | 1.369 | 0.0000393 |
| Cla97C01G011330.1 | Nitrilase | 1.406 | 0.002970745 |
| Cla97C02G041620.1 | peroxisomal adenine nucleotide carrier 1-like | 1.573 | 0.0000619 |
| Cla97C03G066240.1 | IAA-amino acid hydrolase ILR1-like 4 | 1.578 | 0.000195234 |
| Cla97C05G085350.1 | enoyl-CoA delta isomerase 2, peroxisomal-like | 1.483 | 0.008825054 |
| Cla97C07G130770.1 | Glycosyltransferase | 1.614 | 0.003939776 |
| Cla97C09G174190.1 | Glycosyltransferase | 1.542 | 0.00000783 |
| Cla97C09G174200.1 | Glycosyltransferase | 2.569 | 0.001719473 |
| Cla97C10G196500.1 | E3 ubiquitin-protein ligase RGLG2 | 1.394 | 0.002451966 |
| Cla97C09G165630.1 | Hydroxycinnamoyl-CoA shikimate/quinate hydroxycinnamoyltransferase | 1.927 | 0.000158748 |
| Cla97C01G016350.1 | Indole-3-acetic acid-amido synthetase GH3.3, putative | 1.423 | 0.011798393 |
| Cla97C05G089570.1 | Gibberellin 20-oxidase | 3.717 | 0.000131542 |
| Cla97C09G184420.1 | Kaurene synthase-like protein 1 | 1.449 | 0.009161584 |
| Cla97C02G032560.1 | 2-oxoglutarate (2OG) and Fe(II)-dependent oxygenase superfamily protein | 2.539 | 0.000454401 |

Supplementary Data S1. RNA-seq analysis of four critical fruit development stages in watermelon

Supplementary Data S2. TMT-labeled quantitative proteomic analysis of four critical fruit development stages in watermelon

Supplementary Data S3. Mfuzz analysis of DEPs

Supplementary Data S4. GO enrichment analysis of 18DAPvs10DAP

Supplementary Data S5. KEGG pathway enrichment analysis of 18DAPvs10DAP

Supplementary Data S6. GO enrichment analysis of 26DAPvs18DAP

Supplementary Data S7. KEGG pathway enrichment analysis of 26DAPvs18DAP

Supplementary Data S8. GO enrichment analysis of 34DAPvs26DAP

Supplementary Data S9. KEGG pathway enrichment analysis of 34DAPvs26DAP

Supplementary Data S10. Correlation analysis of overlapping mRNAs and proteins

Supplementary Data S11. Quantification of mRNAs and proteins

Supplementary Data S12. GSEA analysis of overlapping mRNAs and proteins

Supplementary Data S13. Hierarchical analysis of mRNAs and proteins

Supplementary Data S14. KEGG pathway enrichment analysis of six clusters from hierarchical analysis
